# Early continuous glucose monitoring-derived glycemic patterns are associated with subsequent insulin resistance and gestational diabetes mellitus development during pregnancy

**DOI:** 10.1186/s13098-024-01508-4

**Published:** 2024-11-14

**Authors:** Chee Wai Ku, Ruther Teo Zheng, Hong Ying Tan, Jamie Yong Qi Lim, Ling-Wei Chen, Yin Bun Cheung, Keith M. Godfrey, Jerry Kok Yen Chan, Fabian Yap, Ngee Lek, See Ling Loy

**Affiliations:** 1 Department of Reproductive Medicine, KK Women’s and Children’s Hospital, 100 Bukit Timah Road, Singapore, 229899 Singapore; 2 Duke-NUS Medical School, 8 College Road, Singapore, 169857 Singapore; 3 Endocrinology Service, Department of Pediatrics, KK Women’s and Children’s Hospital, 100 Bukit Timah Road, Singapore, 229899 Singapore; 4 Yong Loo Lin School of Medicine, National University of Singapore, 10 Medical Drive, Singapore, 117597 Singapore; 5 Lee Kong Chian School of Medicine, Nanyang Technological University, 59 Nanyang Drive, Experimental Medicine Building, Singapore, 636921 Singapore; 6 Institute of Epidemiology and Preventive Medicine, College of Public Health, National Taiwan University, No. 17 Xu-Zhou Road, Taipei, 10055 Taiwan; 7 Master of Public Health Program, College of Public Health, National Taiwan University, No. 17 Xu-Zhou Road, Taipei, 10055 Taiwan; 8 Program in Health Services & Systems Research, Duke-NUS Medical School, 8 College Road, Singapore, 169857 Singapore; 9 Tampere Centre for Child, Adolescent and Maternal Health Research, Tampere University, 33014 Tampere, Finland; 10 Medical Research Council Lifecourse Epidemiology Centre, University of Southampton, Southampton, SO16 6YD UK; 11 National Institute for Health Research Southampton Biomedical Research Centre, University of Southampton and University Hospital Southampton National Health Service Foundation Trust, Southampton, SO16 6YD UK

**Keywords:** Continuous glucose monitoring, Glycemic control/variability, Gestational diabetes mellitus, Insulin resistance

## Abstract

**Background:**

Gestational diabetes mellitus (GDM) and insulin resistance (IR) increase the risk of adverse pregnancy outcomes. We aimed to examine the relationship of interstitial glucose assessed by continuous glucose monitoring (CGM) at early gestation, and the subsequent development of IR and GDM, and to determine 24-h interstitial glucose centile distributions in women with normal (non-IR and non-GDM) and suboptimal glycemic status (IR and/or GDM).

**Methods:**

CGM measurements were taken for 3–10 days at 18–24 weeks’ gestation, followed by fasting serum insulin and oral glucose tolerance testing at 24–28 weeks’ gestation. IR and GDM were determined by the updated Homeostasis Model Assessment of IR score of ≥ 1.22 and 2013 World Health Organization criteria, respectively. Risks of IR and GDM were estimated using modified Poisson models, and hourly interstitial glucose centiles determined using Generalized Additive Models for Location, Scale and Shape.

**Results:**

This prospective cohort study involved 167 pregnant women in Singapore, with a mean age of 31.7 years, body mass index of 22.9 kg/m^2^, and gestation of 20.3 weeks. 25% of women exhibited IR and 18% developed GDM. After confounders adjustment, women with suboptimal glycemic control, indicated by higher mean daily glucose (risk ratio 1.42; 95% confidence interval 1.16, 1.73), glucose management indicator (1.08; 1.03, 1.12), and J-index (1.04; 1.02, 1.06), as well as those with greater glycemic variability, indicated by higher standard deviation (1.69; 1.37, 2.09), coefficient of variation (1.03; 1.00, 1.06), and mean amplitude of glycemic excursions (1.4; 1.14, 1.35) derived from CGM in early gestation were associated with higher risks of developing IR in later gestation. These associations were similarly observed for the development of GDM. Centile curves showed that, compared to those with normal glycemic status, women with suboptimal glycemic status had higher glucose levels, with greater fluctuations throughout 24 h.

**Conclusions:**

In pregnant women who subsequently developed IR and GDM, interstitial glucose levels assessed by CGM were elevated and varied greatly. This supports the potential use of CGM to screen for glycemic changes early in pregnancy.

**Supplementary Information:**

The online version contains supplementary material available at 10.1186/s13098-024-01508-4.

## Background

To ensure sufficient glucose supply for the growing fetus, maternal insulin resistance (IR) increases during pregnancy, especially in the latter half of the second trimester due to placental hormone effects [1, 2]. High IR is associated with increased risk of adverse outcomes, including pre-eclampsia, cardiovascular issues in mothers [3], and large-for-gestational-age births and childhood obesity in offspring [3]. Moreover, elevated IR contributes to gestational diabetes mellitus (GDM) [4] development, which can lead to a range of both short- and long-term complications in mother–child [5]. The prevalence of GDM has been increasing and affects approximately 14% of pregnancies worldwide [6]. GDM is currently diagnosed based on an oral glucose tolerance test (OGTT) between 24 and 28 weeks’ gestation [7]. Studies have shown that glycemic dysregulation early in pregnancy heightens the risks of adverse maternal-child health outcomes [8], highlighting the need for early screening and intervention [9]. However, there are no widely recognized and accepted clinical tools available to detect early gestational glycemic changes prior to the onset of elevated IR or GDM development, which could enable timely intervention.

The use of continuous glucose monitoring (CGM) in women with GDM has been explored increasingly in recent years [10]. Compared to OGTT which only measures glucose tolerance at a single time point during pregnancy, CGM provides a dynamic overview of glucose fluctuations throughout 24 h. Hence, using CGM to identify glycemic control at earlier stages of pregnancy may facilitate timely interventions to prevent poor glycemic outcomes in later pregnancy. Indeed, studies have demonstrated that women with GDM exhibited poorer glycemic control and glycemic variability as assessed through CGM [10–15]. However, even though current guidelines have shed light on the glycemic targets for pregnant women with type one diabetes mellitus [16], there is a lack of evidence for CGM targets in women with normal pregnancies. Furthermore, these studies had either a cross-sectional or case–control design, and only assessed CGM-derived parameters after GDM diagnosis. Therefore, it remains uncertain if the poorer glycemic control and glycemic variability precedes GDM diagnosis and if these parameters can be used in early pregnancy to screen for subsequent GDM diagnosis. There has only been one previous study that assessed the use of CGM-derived parameters as a possible predictor of subsequent GDM diagnosis. This prospective observational study conducted in Singapore reported higher CGM-derived glycemic variability indices in the first and second trimesters in women who were subsequently diagnosed with GDM in the third trimester [17]. However, the study was constrained by a small sample size and limited GDM cases.

In this study with a larger dataset, we aimed to determine the potential use of CGM glycemic patterns as an early screening tool to identify pregnant women at-risk of developing abnormal glycemic states (IR and/or GDM). We hypothesized that interstitial glucose levels, assessed by CGM, will be elevated, and display significant variability over 24 h in pregnant women who will subsequently develop IR and GDM. To test this hypothesis, we sought (i) to examine the relationships of CGM-derived interstitial glucose measurements assessed at mean 20 weeks’ gestation with IR and GDM status ascertained at a mean of 25 weeks’ gestation, and (ii) to determine 24-h interstitial glucose centile distributions in women with normal (non-IR and non-GDM) and suboptimal (IR and/or GDM) glycemic status.

## Methods

### Study design

We used data from a prospective cohort study designed to examine nocturnal eating pattern and glucose metabolism among pregnant women (NCT03803345) [18]. The study was conducted at KK Women’s and Children’s Hospital (KKH), Singapore, and recruitment took place between March 2019 and October 2021. The study adhered to the principles outlined in the Declaration of Helsinki and the findings were reported following the Strengthening the Reporting of Observational Studies in Epidemiology guidelines [19].

### Ethical approval

The Centralized Institutional Review Board of SingHealth approved the study (Reference 2018/2529). All participants provided written informed consent.

### Participants

Women were eligible for the study if they were between 18- and 24-weeks’ gestation, aged 18 years and above, had Singapore citizenship or Singapore permanent residence status. We excluded women who were diagnosed with GDM at recruitment, had pre-existing Type 1 or 2 diabetes, were on routine night-shift work, used anticonvulsant medications or oral steroids in the past month, had known or suspected allergy to medical grade adhesives. Additionally, women who were diagnosed with chronic kidney disease, preeclampsia, or had multiple pregnancies were not included due to the lack of evidence supporting the accuracy of the CGM device (Freestyle Libre Pro, Abbott, Germany) during pregnancy.

### Study procedures and data collection

The study procedures, detailed elsewhere [18], involved a baseline assessment between 18 and 24 weeks gestation. This included collection of data on sociodemographic characteristics (age, ethnicity, education), physical activity (assessed by the International Physical Activity Questionnaire-Short Form [20], allowing calculation of metabolic equivalent of task score [MET-min]), meal regularity (frequency of skipped and/or delayed meals per week) [21], history of GDM, and family diabetes history. Pre-pregnancy body mass index (BMI) was calculated using self-reported weight (kg) divided by height squared (m^2^) (measured by the SECA 213 stadiometer, Germany). Participants were fitted with a blind CGM sensor (Freestyle Libre Pro, Abbott, Germany) on the posterior upper arm, recording interstitial glucose levels every 15 min for up to 10 days. At 24 to 28 weeks’ gestation, participants underwent a 3-point (0, 1-, and 2-h) 75-g OGTT. Plasma glucose and fasting insulin were assessed using the Abbott Alinity c glucose enzymatic (Hexokinase) assay (Germany) and the Abbott Alinity insulin immunochemiluminometric assay (Germany), respectively. Blood samples were analyzed within one hour of collection at the KKH laboratory, following standardized clinical protocols.

### Assessment of CGM parameters

We downloaded the interstitial glucose values from the LibreView software and used EasyGV (version 8) [22] to derive glycemic control and glycemic variability indices from at least 3 complete 24-h CGM readings. The glycemic control indices included mean daily glucose (mmol/L), glucose management indicator (GMI; mmol/mol) [23], J-index [24], percentage of time in range 3.5–7.8 mmol/L (TIR) [25], percentage of time above the target range 7.8 mmol/L (TAR) [25], and percentage of time below the target range 3.5 mmol/L (TBR) [25]. In this study, the term mean daily glucose is used to refer to mean interstitial glucose levels measured by CGM. The glycemic variability indices included standard deviation (SD; mmol/L), coefficient of variation (CV; %), and mean amplitude of glucose excursions (MAGE; mmol/L). MAGE quantifies blood glucose variability by calculating the average of significant upward or downward excursions that surpass a defined threshold [26–29]. This threshold is determined by the SD of blood glucose over a 24-h period.

### Assessment of IR and GDM

Insulin resistance is determined using the HOMA calculator [30] where we categorized participants with IR if they had an updated Homeostasis Model Assessment for IR (HOMA2-IR) [30] score of at least 1.22, following cutoff points for prediabetes [31]. GDM diagnosis was based on the 2013 World Health Organization criteria [32]: fasting glucose ≥ 5.1 mmol/L, or 1-h glucose ≥ 10.0 mmol/L, or 2-h glucose ≥ 8.5 mmol/L. When OGTT results were unavailable, we retrieved GDM diagnosis from delivery records.

### Statistical analysis

We performed statistical analyses using Stata version 16 (StataCorp LLC, College Station, TX) and R programming (R Foundation for Statistical Computing, Vienna, Austria). We compared baseline characteristics and CGM-derived glycemic values of participants based on their IR and GDM status using an independent t-test or Pearson’s Chi-squared test, as appropriate. In addition, we further compared the CGM values according to baseline characteristics. The CGM values were log(e)-transformed and presented in geometric mean, with 95% confidence intervals (CI).

We applied modified Poisson regression models with covariate adjustment to examine the associations of glycemic control and glycemic variability indices derived from CGM with the risk of IR and GDM. Results are presented as risk ratios (RRs) and 95% CI. Models were adjusted for age (continuous), ethnicity (Chinese vs. non-Chinese), years of education (continuous), parity (nulliparous vs. multiparous), history of GDM or family history of diabetes (no vs. yes), pre-pregnancy BMI (continuous), irregular meal (no < 3 times vs. yes ≥ 3 times skipped or delayed meals per week), and physical activity (< 600 vs. ≥ 600 MET-min/week) [33], These potential confounders were identified from previous literature [33–35] and based on the disjunctive cause criteria, [36] guided by a directed acyclic graph. We used Stata to implement the models.

We used generalized estimating equations (GEE) with an exchangeable covariance structure and an identity link function to examine the differences in predicted mean estimates of median interstitial glucose levels over 24 h based on glycemic status. GEE accounts for the non-independence of multiple interstitial glucose measurements. In the GEE model, we treated glycemic status as a covariate and adjusted for the same variables as in the modified Poisson models, plus an interaction term between glycemic status and time. Additionally, we performed a sensitivity analysis by including only participants with complete 10-day 24-h CGM readings. The interstitial glucose values were log_e_-transformed before analyses. Effect estimates are presented as geometric mean ratios (GMRs), with respective 95% CI. We used the geepack package in R [37–39] to implement the models.

To better understand the distribution of interstitial glucose levels by glycemic status, we fitted centile curves for the CGM readings against time in participants with normal (non-IR and non-GDM) and suboptimal (IR and/or GDM) glycemic status, using the generalized additive model for location, scale and shape (GAMLSS) [40] at the 2.5th, 5th, 25th, 50th, 75th, 95th and 97.5th percentile. Within the GAMLSS framework, we modelled predicted glucose levels as a non-linear function of time using the Box-Cox t (BCT) distribution [40] with four parameters: mu (μ), the median of the distribution; sigma (σ), approximately the coefficient of variation; nu (ν), which controls for skewness; and tau (τ), which controls for the kurtosis of the distribution. We used the gamlss package in R [40] to derive the curves.

## Results

### Participant characteristics

Of the 300 women enrolled, 172 women completed at least 3 days of CGM at 18–24 weeks gestation with an average of 828 CGM readings (200 to 1217 readings). After excluding five women without GDM status data and nine without IR status data, the final sample sizes were 167 for the GDM outcome analysis and 158 for the IR outcome analysis, respectively (Additional file 1). There were 112 women (67.1%) with at least 10 days of CGM data. 40 women out of 158 (25.3%) had IR, 30 out of 167 (18.0%) had GDM, and 62 out of 167 (37.1%) had suboptimal glycemic status (IR and/or GDM). Women with IR had fewer years of education (14.0 vs. 14.5 years) and a higher BMI before pregnancy (25.3 vs 22.0 kg/m^2^) than their non-IR counterparts. There were no significant differences in baseline characteristics between GDM and non-GDM women (Table 1). When comparing characteristics of included (n = 167) and excluded women (n = 133), included women were older (31.7 vs. 30.3 years) and had higher years of education (14.7 vs. 13.8 years) (Additional file 2).

**Table 1:** Baseline characteristics of pregnant women by glycemic status

Characteristics	Total (n = 167)	Non-IR (n = 118)	IR (n = 40)	p	Non-GDM (n = 137)	GDM (n = 30)	p
Gestation at enrolment, weeks	20.3 ± 0.5	20.3 ± 0.4	20.4 ± 0.8	0.393	20.31 ± 0.5	20.5 ± 0.8	0.293
Age, years	31.7 ± 4.2	32.1 ± 4.3	30.8 ± 3.7	0.063	31.5 ± 4.1	32.8 ± 4.5	0.152
Ethnicity, n (%)				0.541			0.781
Chinese	142 (85.0)	102 (86.4)	33 (82.5)		116 (84.7)	26 (86.7)	
Non-Chinese	25 (15.0)	16 (13.6)	7 (17.5)		21 (15.3)	4 (13.3)	
Education, years	14.7 ± 2.2	14.5 ± 2.3	14.0 ± 1.9	0.012	14.7 ± 2.1	14.6 ± 3.0	0.766
Parity, n (%)				0.870			0.455
Nulliparous	107 (64.1)	75 (63.6)	26 (65.0)		86 (62.8)	21 (70.0)	
Multiparous	60 (35.9)	43 (36.4)	14 (35.0)		51 (37.2)	9 (30.0)	
History of GDM or family history of diabetes, n (%)				0.711			0.864
No or not applicable	126 (75.4)	88 (74.6)	31 (77.5)		103 (75.2)	23 (76.7)	
Yes	41 (24.6)	30 (25.4)	9 (22.5)		34 (24.8)	7 (23.3)	
Pre-pregnancy BMI, kg/m^2^	22.9 ± 3.9	22.0 ± 3.4	25.3 ± 4.2	< 0.001	22.8 ± 4.0	23.5 ± 3.7	0.335
Irregular meal, n (%)				0.131			0.864
No	130 (77.8)	96 (81.4)	28 (70.0)		107 (78.1)	23 (76.7)	
Yes	37 (22.2)	22 (18.6)	12 (30.0)		30 (21.9)	7 (23.3)	
Physical activity, n (%)				0.200			0.338
Active (≥ 600 MET-min/week)	123 (73.7)	82 (69.5)	32 (80.0)		103 (75.2)	20 (66.7)	
Inactive (< 600 MET-min/week)	44 (26.3)	36 (30.5)	8 (20.0)		34 (24.8)	10 (33.3)	

Continuous data are presented in mean ± SD and categorical data are presented in frequency and percentages. Nine women without IR status data were excluded. *IR* insulin resistance based on HOMA2-IR at least 1.22. GDM, gestational diabetes mellitus based on 2013 WHO criteria, *BMI* body mass index, *MET* metabolic equivalent of task

p-value derived from independent t-test or Pearson’s Chi-squared test, as appropriate

### CGM-derived glycemic measures, IR and GDM development

Glycemic control and glycemic variability indices derived from CGM were assessed at a mean of 20 weeks’ gestation, according to IR and GDM status ascertained at a mean of 25 weeks’ gestation (Table 2). Women with IR had poorer glycemic control, indicated by higher mean daily glucose levels, GMI, and J-index, as well as lower TBR (all p < 0.05). Women with GDM had poorer glycemic control, indicated by higher mean daily glucose, GMI, J-index, TAR, and lower TBR; these women also had a greater glycemic variability, indicated by higher SD, CV, and MAGE (all p < 0.05). After adjustment for socio-demographic and lifestyle factors, the majority of glycemic control and glycemic variability indices remained associated with the risks of IR and GDM. In particular, mean daily glucose and SD were associated with the highest risk of IR (1.42; 95% CI 1.16, 1.73, and 1.69; 95% CI 1.37, 2.09 respectively) and GDM (1.84; 95% CI 1.45, 2.33, and 2.37; 95% CI 1.66, 3.38 respectively) (Table 3). When comparing with baseline characteristics, the non-Chinese women have a lower TIR but higher TBR than Chinese women and women who skipped/delayed at least 3 meals per week have lower mean daily glucose, J-index, TIR and higher TBR than women with regular meals (all p < 0.05). (Additional file 3).

**Table 2:** Comparison of CGM-derived glycemic control and variability indices in pregnant women based on glycemic status

CGM Measures	Total (n = 167)	Non-IR (n = 118)	IR (n = 40)	p	Non-GDM (n = 137)	GDM (n = 30)	p
**Glycemic control index**							
Mean daily glucose, mmol/L	4.46 (4.37, 4.56)	4.35 (4.26, 4.43)	4.84 (4.57, 5.12)	0.003	4.35 (4.27, 4.43)	5.02 (4.69, 5.36)	0.002
GMI, mmol/mol	33.79 (33.34, 34.24)	33.21 (32.82, 33.61)	35.60 (34.25, 36.99)	0.003	33.23 (32.86, 33.61)	36.44 (34.79, 38.16)	0.002
J-index	9.86 (9.43, 10.32)	9.29 (8.93, 9.67)	11.68 (10.21, 13.36)	0.017	9.26 (8.92, 9.61)	13.17 (11.32, 15.31)	0.006
TIR, %	77.82 (74.74, 81.02)	76.86 (73.36, 80.51)	81.91 (74.55, 89.99)	0.073	77.63 (74.42, 80.97)	78.68 (69.67, 88.86)	0.587
TAR, %	0.93 (0.72, 1.21)	0.74 (0.56, 0.97)	1.52 (0.77, 3.01)	0.092	0.64 (0.50, 0.82)	2.70 (1.57, 4.65)	0.033
TBR, %	10.83 (8.96, 13.09)	12.34 (9.86, 15.44)	6.50 (4.43, 9.53)	< 0.001	11.64 (9.50, 14.27)	7.32 (4.37, 12.26)	0.003
Glycemic variability index							
SD, mmol/L	1.03 (1.00, 1.08)	0.99 (0.96, 1.03)	1.13 (1.01, 1.27)	0.056	0.98 (0.95, 1.01)	1.33 (1.18, 1.50)	0.002
CV, %	23.19 (22.47, 23.92)	22.88 (22.09, 23.69)	23.44 (21.64, 25.39)	0.457	22.52 (21.83, 23.24)	26.46 (24.23, 28.90)	0.009
MAGE, mmol/L	2.70 (2.60, 2.81)	2.62 (2.51, 2.72)	2.93 (2.62, 3.27)	0.080	2.56 (2.47, 2.65)	3.46 (3.07, 3.90)	0.002

Data are presented as geometric mean (95% confidence interval). Nine women without IR status data were excluded. CGM, continuous glucose monitoring; IR, insulin resistance based on HOMA2-IR at least 1.22; *GDM* gestational diabetes mellitus based on 2013 WHO criteria, *GMI*, glucose management indicator, *TIR* percentage of time in range 3.5–7.8 mmol/L, *TAR* percentage of time above target range 7.8 mmol/L, *TBR* percentage of time below target range 3.5 mmol/L, *SD* standard deviation, *CV* coefficient of variation, *MAGE* mean amplitude of glycemic excursions

p-value derived from independent t-test from log-transformed CGM-derived glycemic indices

**Table 3:** Associations between CGM-derived glycemic control and variability indices with risk of IR and GDM (n = 167)

CGM Measures	Risk Ratio (95% CI)			
	IR		GDM	
	Model 1 (crude)	Model 2 (adjusted)	Model 1 (crude)	Model 2 (adjusted)
Glycemic control index				
Mean daily glucose, mmol/L	1.42 (1.21, 1.66)	1.42 (1.16, 1.73)	1.62 (1.33, 1.96)	1.84 (1.45, 2.33)
GMI, mmol/mol	1.08 (1.04, 1.11)	1.08 (1.03, 1.12)	1.11 (1.06, 1.15)	1.14 (1.08, 1.20)
J-index	1.04 (1.03, 1.06)	1.04 (1.02, 1.06)	1.06 (1.04, 1.08)	1.07 (1.05, 1.09)
TIR, %	1.01 (1.00, 1.03)	1.01 (1.00, 1.03)	1.01 (0.99, 1.03)	1.00 (0.98, 1.03)
TAR, %	1.02 (1.01, 1.03)	1.02 (1.01, 1.03)	1.03 (1.02, 1.04)	1.04 (1.02, 1.06)
TBR, %	0.98 (0.96, 0.99)	0.98 (0.96, 0.99)	0.97 (0.94, 1.00)	0.97 (0.94, 0.99)
Glycemic variability index				
SD, mmol/L	1.62 (1.32, 1.99)	1.69 (1.37, 2.09)	2.21 (1.63, 3.01)	2.37 (1.66, 3.38)
CV, %	1.02 (0.99, 1.05)	1.03 (1.00, 1.06)	1.06 (1.04, 1.09)	1.07 (1.04, 1.10)
MAGE, mmol/L	1.20 (1.11, 1.31)	1.24 (1.14, 1.35)	1.39 (1.21, 1.58)	1.42 (1.23, 1.65)

Data were analyzed using modified Poisson regression models to examine the associations between CGM indices with IR and GDM. Models 2 were adjusted for age, ethnicity, years of education, parity, history of GDM or family history of diabetes, pre-pregnancy body mass index, irregular meal, and physical activity. *CI* confidence interval, *IR* insulin resistance based on HOMA2-IR at least 1.22, *GDM* gestational diabetes mellitus based on 2013 WHO criteria, *GMI* glucose management indicator, *TIR* percentage of time in range 3.5–7.8 mmol/L, *TAR* percentage of time above target range 7.8 mmol/L, *TBR* percentage of time below target range 3.5 mmol/L, *SD* standard deviation, *CV* coefficient of variation, *MAGE* mean amplitude of glycemic excursions

**Fig. 1:**
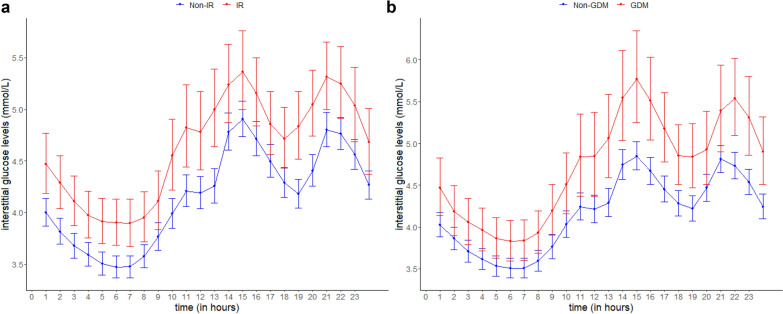
The predicted 24-h interstitial glucose levels for women by (**a**) IR (GMR 1.12; 95% CI 1.05, 1.19) and (**b**) GDM status (1.11; 1.03, 1.20) based on the GEE analysis. Red represents the women with IR or GDM and blue represents the women with non-IR or non-GDM. The circle markers and capped vertical lines represent the predicted mean daily glucose levels and the respective 95% CI based on the exponentiated log-transformed hourly median glucose values. Models were adjusted for age, ethnicity, years of education, parity, history of GDM or family history of diabetes, pre-pregnancy body mass index, irregular meal, physical activity, and an interaction term between glycemic status and time. *CI* confidence intervals, *GDM* gestational diabetes mellitus based on 2013 World Health Organization criteria, *GEE* generalized estimating equations, *GMR* geometrical mean ratio, *IR* insulin resistance based on HOMA2-IR of at least 1.22

When interstitial glucose levels over a 24-h period were compared between women according to their glycemic status, those with IR showed 12% higher glucose levels (GMR 1.12; 95% CI 1.05, 1.19), equivalent to a difference of 0.45 mmol/L, than their counterparts without IR, after adjustment for potential confounders. Similarly, women with GDM showed 11% higher glucose levels over 24 h (1.11; 1.03, 1.20), equivalent to a difference of 0.46 mmol/L, compared to those without GDM. When the hourly adjusted means of the median interstitial glucose levels were plotted throughout 24 h, consistently higher glucose levels were observed in women who later developed IR or GDM compared to their normal counterparts (Fig. 1). When analysis was restricted to women with complete 10 day 24-h CGM readings (n = 112), women with IR and GDM respectively showed 12% (1.12; 1.03, 1.21) and 9% (1.09; 0.99, 1.20) higher glucose levels across 24 h than their normal counterparts (Additional file 4).

### 24-h interstitial glucose centile distributions in women with normal and suboptimal glycemic measures

**Fig. 2:**
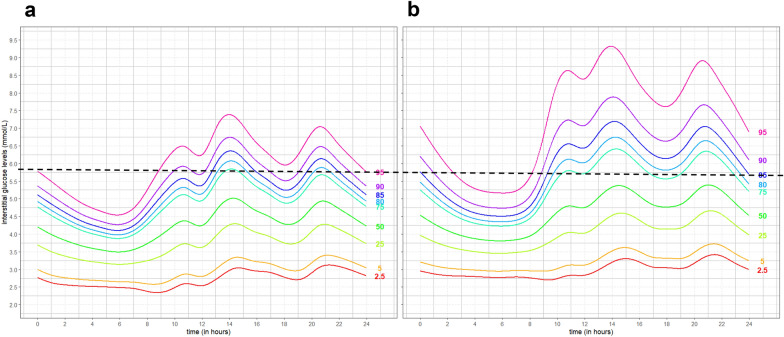
The centile curves of 24-h interstitial glucose levels from Box-Cox transformation among pregnant women with (**a**) non-IR and non-GDM, and (**b**) IR and/or GDM. A line across the 95th centile for pregnant women without IR and GDM corresponds to the 85th centile among those with IR and/or GDM, which suggests that those above the 95th centile are three times more likely to have IR and/or GDM. GDM, gestational diabetes mellitus based on 2013 World Health Organization criteria; IR, insulin resistance based on HOMA2-IR of at least 1.22

Centile curves of the interstitial glucose readings for women with normal and suboptimal glycemic status were calculated using the BCT models (Fig. 2). These curves illustrate the higher glucose levels and greater fluctuations throughout 24 h in women with suboptimal glycemic status when compared to those with normal glycemic status. The centile curves demonstrated a distinct diurnal pattern of interstitial glucose levels for both groups of women. From midnight onwards, interstitial glucose levels gradually declined, reaching their lowest point at around 6 am. Starting from 7 am, there was a noticeable increase in glucose levels, peaking between 11 am and 2 pm. The glucose levels decreased until 7 pm, at which point they began to rise again, reaching a second peak at around 9 pm. In the subsequent hours, there was a tapering off as the levels decreased, aligned with the onset of the nocturnal phase. The 95th centile among pregnant women without IR and GDM corresponds to the 85th centile among those with IR and/or GDM. Thus, above this cutoff, pregnant women are three times more likely to have IR and/or GDM.

## Discussion

### Main findings

This study has shown that women who later experienced elevated IR or developed GDM exhibited higher pre-diagnosis interstitial glucose levels with greater variability throughout the 24-h period. The generated centile curves similarly showed elevated levels and variability in interstitial glucose among women with suboptimal glycemic status, setting them apart from those with normal status, even before IR or GDM diagnosis. These findings support the existing evidence advocating early CGM screening for subsequent glucose dysregulation and identify pregnant women at risk of developing elevated IR or GDM in later stages of pregnancy. Notably, most baseline characteristics, such as pre-pregnancy BMI, maternal age, parity, history of GDM or family history of diabetes, are not significantly associated with CGM parameters.

### Comparison with other studies

We provide new evidence showing poorer glycemic control assessed by CGM in earlier gestation among women with elevated IR in later gestation. We also demonstrate poorer glycemic control in early gestation among women subsequently diagnosed with GDM. The only study which examined the prospective association between CGM-derived glycemic control and subsequent development of GDM showed a non-significant association [17]. However, in a previous observational study of pregnant women already diagnosed with GDM, mean daily glucose was reported to be higher compared to healthy pregnant women between 24 and 36 weeks of gestation using the flash glucose monitoring system [11]. Another study reported significant associations only in the second trimester, but not the third [12], while other studies reported null associations [25, 41, 42]. Our study was able to demonstrate the utility of using CGM to monitor glycemic control levels in pregnancy and address the gap in literature with regard to the prospective association of glycemic control with IR and GDM development. In the hyperglycemia and adverse pregnancy outcomes (HAPO) study, it was reported that there was a strong and continuous relationship between maternal blood glucose level and adverse maternal and neonatal outcomes [43]. Hence, monitoring glycemic control levels earlier in pregnancy using CGM may pave the way for earlier interventions and improve glycemic parameters, and thus maternal and neonatal outcomes. Notably, TBR was elevated in patients without IR or GDM at 12.3% and 11.6% respectively. Our findings were similar to a local observational study [17] with a smaller sample size of 39, which utilized early pregnancy CGM measurement in the first and second trimester to predict GDM diagnosis. In that study, the patients without GDM had a higher TBR of 30.2% compared to their GDM counterpart with a TBR of 26.9%. Currently, there is a lack of evidence on CGM targets including TBR for women with normal pregnancy [16]. However, other studies have suggested that more stringent targets [44] and closer monitoring of overnight glucose profiles may be required to improve outcomes in pregnant women with GDM [45].

At present, little is known about the association between glycemic variability and IR in pregnant women. This study provides new evidence, showing that women exhibiting greater glycemic variability are at an increased risk of developing IR. Our findings also reveal increased glycemic variability in women later diagnosed with GDM. This aligns with a prospective observational study conducted in Singapore in 2022, which identified higher first and second trimester glycemic variability indices (SD and MAGE) in women subsequently diagnosed with GDM, compared to those without a diagnosis [17]. However, it is noteworthy that in that study, the associations of glycemic variability indices with GDM development were inconsistent, perhaps as a result of the small sample size (n = 60). In contrast, our study consistently demonstrated universally elevated glycemic variability indices in women subsequently diagnosed with GDM. Other case–control studies also showed higher MAGE levels in pregnant women who were diagnosed with GDM than those who were not [10, 12]. Monitoring glycemic variability is especially important in pregnancy due to the established association of poorer glycemic variability with poorer pregnancy outcomes [46]. Poor glycemic variability as indicated by elevated MAGE has been associated with adverse maternal and neonatal outcomes, including large or small for gestational age, higher birth weight, and neonatal hypoglycemia [47]. As such, this study offers much-needed evidence supporting the use of CGM to monitor glycemic variability in early pregnancy and its association with subsequent development of IR and GDM.

The 24-h interstitial glucose centile curves showed higher mean daily glucose levels at all time points of the day and greater glucose fluctuations in women with suboptimal glycemic status (IR and/or GDM). The greatest glucose fluctuation was observed to be during the day, followed by a physiological dip in blood glucose level at night. This physiological dip is consistent with the effects of the circadian rhythm on glucose homeostasis as the metabolic demand of the body is lower during sleep. Similarly, a diurnal elevation can be attributed to factors such as increased food intake, physical activity, and other metabolic processes that are active during waking hours [48]. As such, this variation prompts the development of different blood glucose threshold levels for day and night to allow patients and their physicians to monitor glycemic control and glycemic variability. This may facilitate time-specific lifestyle interventions or medications when glycemic levels are poor. In addition, CGM measures were more clearly differentiated between GDM and non-GDM but not so clearly between IR and non-IR. It is plausible that the greater degree of overlap between non-IR and IR women is due to the compensatory hyperinsulinemia in IR women which helps to maintain lower interstitial glucose levels, compared to the decompensated status of those who developed GDM.

Additionally, the development of these centile curves provides a proof of concept for its potential utility as a reference curve to screen for abnormal glycemic regulations in early gestation. This paves the way for the comparison of an individual’s interstitial glucose levels to those of a reference population. Using the 95th centile for pregnant women without IR and GDM, levels above which corresponds to a three-fold increase in risk of IR and/or GDM. This provides a theoretical basis for future studies to validate the use of standardized centile curves for triaging and monitoring women with normal or suboptimal glycemic status. Early screening and intervention is essential to reduce the potential adverse health outcomes to both mother and child [9]. Hence, development of such standardized curves could potentially facilitate early pregnancy screening and interventions to reduce the adverse outcomes associated with suboptimal glycemic status.

## Strengths and limitations

A major strength in our study lies in its prospective design, which enabled us to assess the associations between CGM-derived data at 18 to 24 weeks gestation and subsequently diagnosed IR and/or GDM at 24 to 28 weeks. To the best of our knowledge, this is the first study examining the association between CGM-derived data and subsequent development of IR in pregnant women. This is also the first study to have generated centile curves which could potentially differentiate between normal individuals and those at risk of developing IR and/or GDM later in pregnancy. While our study benefits from a comparatively larger sample size for women undergoing CGM than those reported in previous studies [12, 17], we acknowledge the necessity of expanding the sample size in future studies to enhance the generalizability and facilitate subgroup analyses. The association of fasting intervals, particularly from the perspective of time-restricted feeding, with CGM parameters was not explored in this study. Given the potential importance of such data, we recommend this as a promising direction for future research to gain deeper insights into how specific eating-fasting intervals influence glycemic trends and pregnancy outcomes. Furthermore, we were unable to consider maternal weight gain during pregnancy in the analysis due to incomplete data collected. While gestational weight gain has been reported as a risk factor for increased GV at late pregnancy in women with GDM, its effects in the early pregnancy remain less clear [25].

## Conclusions

Taken together, higher mean daily glucose over 24 h as well as poorer glycemic control and glycemic variability in early gestation are associated with subsequent diagnosis of IR and GDM. The CGM-derived glycemic control and glycemic variability parameters along with the 24-h interstitial glucose centile curves demonstrate the potential utility of CGM as a tool in early pregnancy to screen for subsequent suboptimal glycemic status. This paves the way for early initiation of lifestyle interventions to improve glycemic regulation and reduce the risk of adverse maternal and neonatal outcomes.

## Supplementary Information

Additional file 1. Participant recruitment

Additional file 2. Baseline characteristics of women included and excluded from the study

Additional file 3. Comparison of CGM-derived glycemic control and variability indices in pregnant women based on baseline characteristics

Additional file 4. The predicted 24-hour interstitial glucose levels for women with at least 10 days of CGM readings by (a) IR (GMR 1.12; 95% CI 1.03, 1.21) and (b) GDM status (1.09; 0.99, 1.20) based on the GEE analysis. Red represents the women with IR or GDM and blue represents the women with non-IR or non-GDM. The circle markers and capped vertical lines represent the predicted mean daily glucose levels and the respective 95% CI based on the exponentiated log-transformed hourly median glucose values. Models were adjusted for age, ethnicity, years of education, parity, history of GDM or family history of diabetes, pre-pregnancy body mass index, irregular meal, physical activity, and an interaction term between glycemic status and time. *CI* confidence intervals, *GDM* gestational diabetes mellitus based on 2013 World Health Organization criteria, *GEE* generalized estimating equations, *GMR* geometrical mean ratio, *IR* insulin resistance based on HOMA2-IR of at least 1.22.

## Data Availability

The data that support the findings of this study are available from the corresponding author upon reasonable request.

## Abbreviations

GDM: Gestational diabetes mellitus
IR: Insulin resistance
CGM: Continuous glucose monitoring
OGTT: Oral glucose tolerance test
MET: Metabolic equivalent of task
BMI: Body mass index
GMI: Glucose management indicator
TIR: Time in range
TAR: Time above range
TBR: Time below range
SD: Standard deviation
CV: Coefficient of variation
MAGE: Mean amplitude of glucose excursions
HOMA2-IR: Homeostasis Model Assessment for IR
RR: Risk ratios
CI: 95% Confidence interval
GEE: Generalized estimating equations
GMR: Geometric mean ratios
GAMLSS: Generalized additive model for location, scale and shape
BCT: Box-Cox t
