# Supplementary material for: Early continuous glucose monitoring-derived glycemic patterns are associated with subsequent insulin resistance and gestational diabetes mellitus development during pregnancy
Source: Diabetol Metab Syndr. 2024 Nov 14;16:271. doi: 10.1186/s13098-024-01508-4 (PMC11562738; doi:10.1186/s13098-024-01508-4)
Supplement: Supplementary file 1 — Additional file 1. Participant recruitment [file 13098_2024_1508_MOESM1_ESM.pptx]

## Slide 1
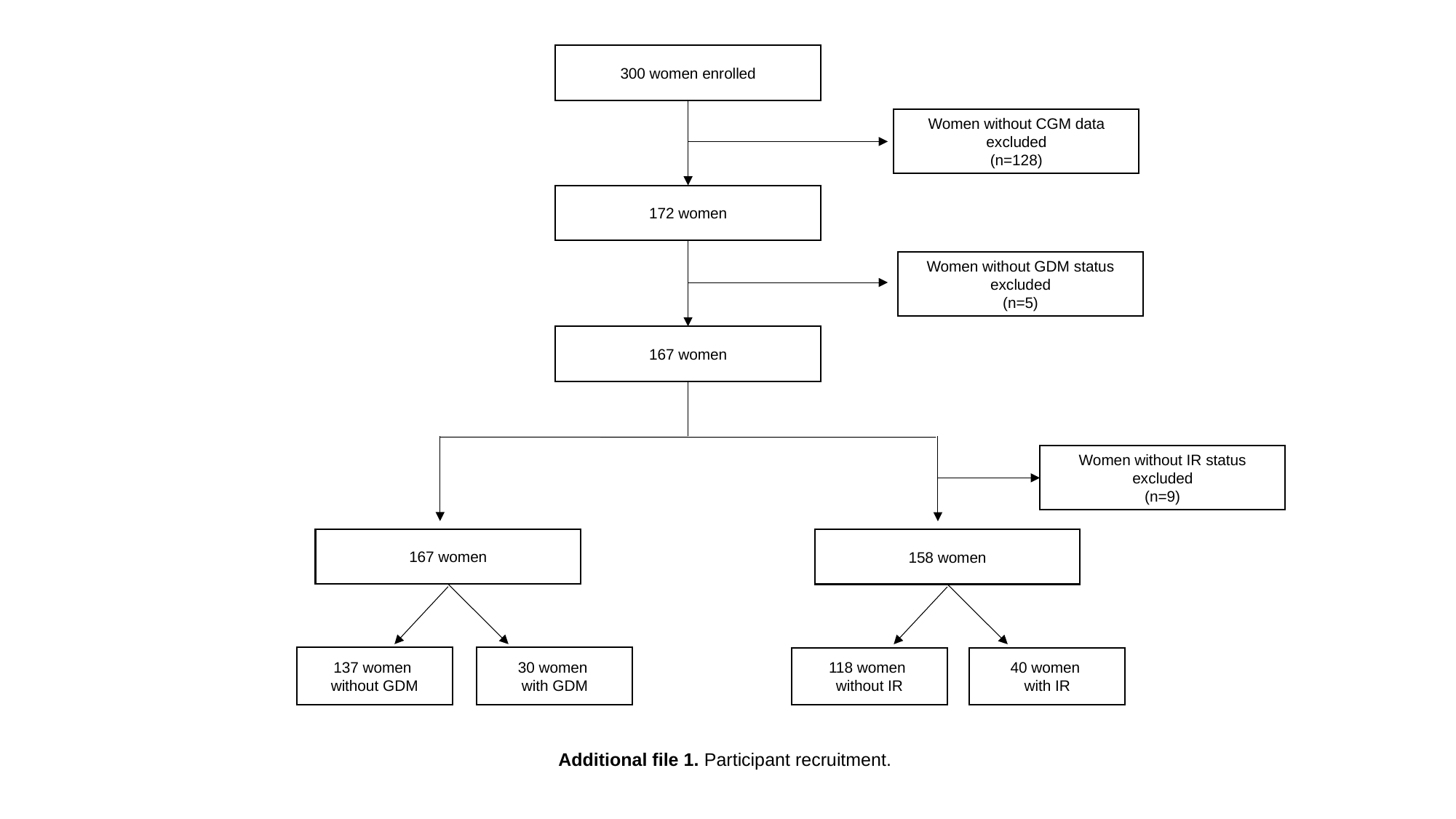

300 women enrolled
Women without CGM data excluded
(n=128)
172 women
Women without GDM status excluded
(n=5)
167 women
Women without IR status
excluded
(n=9)
167 women
158 women
137 women
without GDM
30 women
with GDM
118 women
without IR
40 women
with IR
Additional file 1. Participant recruitment.
