# Supplementary material for: Early continuous glucose monitoring-derived glycemic patterns are associated with subsequent insulin resistance and gestational diabetes mellitus development during pregnancy
Source: Diabetol Metab Syndr. 2024 Nov 14;16:271. doi: 10.1186/s13098-024-01508-4 (PMC11562738; doi:10.1186/s13098-024-01508-4)
Supplement: Supplementary file 2 — Additional file 2. Baseline characteristics of women included and excluded from the study [file 13098_2024_1508_MOESM2_ESM.docx]

### Additional file 2. Baseline characteristics of women included and excluded from the study

| **Characteristics** | **Total (n=300)** | **Excluded (n=133)** | **Included (n=167)** | **p** |
| --- | --- | --- | --- | --- |
| Gestation at enrolment, weeks | 20.4 ± 0.5 | 20.4 ± 0.5 | 20.3 ± 0.5 | 0.488 |
| Age, years | 31.1 ± 4.2 | 30.3 ± 4.2 | 31.7 ± 4.2 | 0.004 |
| Ethnicity, n (%) |  |  |  | 0.126 |
| Chinese | 246 (82.0) | 104 (78.2) | 142 (85.0) |  |
| Non-Chinese | 54 (18.0) | 29 (21.8) | 25 (15.0) |  |
| Education, years | 14.3 ± 2.5 | 13.8 ± 2.8 | 14.7 ± 2.2 | 0.002 |
| Parity |  |  |  | 0.573 |
| Nulliparous | 188 (62.7) | 81 (60.9) | 107 (64.1) |  |
| Multiparous | 112 (37.3) | 52 (39.1) | 60 (35.9) |  |
| History of GDM or family history of diabetes, n (%) |  |  |  | 0.232 |
| No or not applicable | 234 (78.0) | 108 (81.2) | 126 (75.4) |  |
| Yes | 66 (22.0) | 25 (18.8) | 41 (24.6) |  |
| Pre-pregnancy BMI, kg/m² | 22.9 ± 4.1 | 22.8 ± 4.4 | 22.9 ± 3.9 | 0.778 |
| Irregular meal, n (%) |  |  |  | 0.066 |
| No | 221 (73.7) | 91 (68.4) | 130 (77.8) |  |
| Yes | 79 (26.3) | 42 (31.6) | 37 (22.2) |  |
| Physical activity, n (%) |  |  |  | 0.995 |
| Active (≥600 MET-min/week) | 221 (73.7) | 98 (73.7) | 123 (73.7) |  |
| Inactive (<600 MET-min/week) | 79 (26.3) | 35 (26.3) | 44 (26.3) |  |

Continuous data are presented in mean +/- standard deviation and categorical data are presented in frequency and percentages. Chi-squared tests for categorical variables and independent t tests for continuous variables were used to compare the two groups. GDM, gestational diabetes mellitus; BMI, body mass index; MET, metabolic equivalent of task.
