# Supplementary material for: Early continuous glucose monitoring-derived glycemic patterns are associated with subsequent insulin resistance and gestational diabetes mellitus development during pregnancy
Source: Diabetol Metab Syndr. 2024 Nov 14;16:271. doi: 10.1186/s13098-024-01508-4 (PMC11562738; doi:10.1186/s13098-024-01508-4)
Supplement: Supplementary file 3 — Additional file 3. Comparison of CGM-derived glycemic control and variability indices in pregnant women based on baseline characteristics [file 13098_2024_1508_MOESM3_ESM.docx]

**Additional file 3.** Comparison of CGM-derived glycemic control and variability indices in pregnant women based on baseline characteristics

| **Variable** | **N (%)** | **Mean, mmol/L** | **GMI, mmol/mol** | **J-index** | **TIR, %** | **TAR, %** | **TBR, %** | **SD, mmol/L** | **CV, %** | **MAGE, mmol/L** |
| --- | --- | --- | --- | --- | --- | --- | --- | --- | --- | --- |
| **Age group** |  |  |  |  |  |  |  |  |  |  |
| <30 years old | 64 (38.3) | 4.39 (4.28, 4.59) | 33.45 (32.84, 34.30) | 9.60 (9.01, 11.40) | 75.69 (73.76, 82.55) | 0.99 (0.64, 2.75) | 11.67 (15.36, 24.55) | 1.03 (0.96, 1.21) | 23.44 (22.42, 25.83) | 2.69 (2.52, 3.15) |
| ≥30 years old | 103 (61.7) | 4.51 (4.41, 4.70) | 34.00 (33.46, 34.83) | 10.03 (9.55, 11.58) | 79.17 (78.18, 84.44) | 0.91 (0.35, 3.57) | 10.32 (13.54, 19.51) | 1.04 (1.01, 1.13) | 23.03 (22.55, 24.33) | 2.71 (2.65, 2.93) |
| **Ethnicity** |  |  |  |  |  |  |  |  |  |  |
| Chinese | 142 (85.0) | 4.51 (4.43, 4.66) | 33.98 (33.57, 34.66) | 10.05 (9.76, 11.50) | 79.72 (79.15, 84.30) | 0.94 (0.72, 3.14) | 9.83 (13.62, 18.62) | 1.04 (1.02, 1.16) | 23.17 (22.77, 24.71) | 2.74 (2.68, 3.02) |
| Non-Chinese | 25 (15.0) | 4.23 (3.99, 4.57) | 32.71 (31.51, 34.20) | 8.84 (7.90, 10.65) | 67.85 (62.60, 79.09)* | 0.90 (-0.49, 3.38) | 18.70 (18.80, 36.37)* | 0.98 (0.91, 1.09) | 23.26 (22.13, 24.84) | 2.53 (2.35, 2.81) |
| **Highest education** |  |  |  |  |  |  |  |  |  |  |
| Below Tertiary | 70 (41.9) | 4.49 (4.34, 4.78) | 33.96 (33.15, 35.20) | 10.08 (9.38, 12.41) | 74.91 (73.46, 81.99) | 1.01 (0.37, 5.26) | 12.21 (15.13, 23.36) | 1.07 (1.02, 1.20) | 23.74 (23.02, 25.31) | 2.75 (2.64, 3.04) |
| Tertiary | 97 (58.1) | 4.44 (4.37, 4.57) | 33.66 (33.26, 34.22) | 9.71 (9.33, 10.85) | 79.98 (78.68, 84.96) | 0.88 (0.64, 1.70) | 9.93 (13.58, 20.06) | 1.01 (0.97, 1.13) | 22.79 (22.16, 24.58) | 2.67 (2.57, 3.00) |
| **Parity** |  |  |  |  |  |  |  |  |  |  |
| Nulliparous | 107 (64.1) | 4.44 (4.37, 4.59) | 33.67 (33.26, 34.29) | 9.78 (9.48, 10.97) | 77.80 (76.82, 83.41) | 0.95 (0.84, 2.13) | 10.76 (14.78, 21.61) | 1.03 (1.00, 1.15) | 23.29 (22.72, 24.98) | 2.71 (2.62, 3.01) |
| Multiparous | 60 (35.9) | 4.50 (4.33, 4.79) | 33.99 (33.10, 35.27) | 10.01 (9.09, 12.49) | 77.85 (75.98, 84.16) | 0.90 (-0.24, 5.30) | 10.95 (13.53, 20.87) | 1.04 (0.98, 1.18) | 23.00 (22.20, 24.69) | 2.69 (2.57, 3.02) |
| **History of GDM or family history of diabetes** |  |  |  |  |  |  |  |  |  |  |
| No or not applicable | 126 (75.4) | 4.40 (4.34, 4.53) | 33.49 (33.13, 34.03) | 9.63 (9.37, 10.78) | 77.53 (76.74, 82.54) | 0.90 (0.74, 1.88) | 11.29 (15.86, 21.87) | 1.03 (1.00, 1.14) | 23.39 (22.90, 24.98) | 2.70 (2.62, 2.98) |
| Yes | 41 (24.6) | 4.66 (4.41, 5.06) | 34.72 (33.45, 36.50) | 10.61 (9.22, 13.81) | 78.71 (76.03, 87.00) | 1.02 (-0.50, 7.59) | 9.38 (10.01, 19.34) | 1.05 (0.98, 1.19) | 22.56 (21.63, 24.32) | 2.73 (2.56, 3.09) |
| **Pre-pregnancy BMI** |  |  |  |  |  |  |  |  |  |  |
| <25 kg/m^2^ | 118 (70.7) | 4.41 (4.34, 4.54) | 33.52 (33.15, 34.08) | 9.62 (9.41, 10.54) | 77.43 (76.53, 82.59) | 0.85 (0.79, 2.02) | 11.15 (15.73, 21.99) | 1.02 (1.00, 1.11) | 23.18 (22.76, 24.53) | 2.66 (2.61, 2.87) |
| ≥25 kg/m^2^ | 49 (29.3) | 4.59 (4.38, 4.95) | 34.43 (33.34, 35.98) | 10.46 (9.25, 13.77) | 78.76 (76.55, 86.26) | 1.20 (-0.43, 6.32) | 10.04 (11.10, 19.66) | 1.07 (0.97, 1.29) | 23.20 (21.85, 25.85) | 2.81 (2.56, 3.37) |
| **Irregular meal** |  |  |  |  |  |  |  |  |  |  |
| No (<3 times skipped or delayed meals per week) | 130 (77.8) | 4.53 (4.45, 4.70) | 34.09 (33.63, 34.83) | 10.13 (9.81, 11.73) | 79.89 (79.20, 84.56) | 0.91 (0.78, 3.49) | 9.68 (13.16, 18.36) | 1.05 (1.02, 1.17) | 23.09 (22.64, 24.65) | 2.73 (2.67, 3.04) |
| Yes (≥3 times skipped or delayed meals per week) | 37 (22.2) | 4.25 (4.11, 4.45)* | 32.76 (32.04, 33.65)* | 8.96 (8.48, 9.96)* | 70.96 (67.39, 80.30)* | 1.09 (0.26, 1.50) | 15.92 (18.55, 31.74)* | 1.00 (0.94, 1.09) | 23.51 (22.36, 25.45) | 2.60 (2.46, 2.83) |
| **Physical activity** |  |  |  |  |  |  |  |  |  |  |
| Active (≥600 MET-min/week) | 123 (73.7) | 4.45 (4.37, 4.63) | 33.73 (33.26, 34.49) | 9.74 (9.39, 11.16) | 77.88 (77.19, 83.05) | 0.84 (0.42, 3.21) | 11.05 (15.00, 20.75) | 1.02 (0.99, 1.10) | 22.83 (22.44, 23.98) | 2.65 (2.60, 2.86) |
| Inactive (<600 MET-min/week) | 44 (26.3) | 4.50 (4.35, 4.72) | 33.94 (33.18, 34.93) | 10.22 (9.27, 12.44) | 77.64 (74.72, 85.35) | 1.20 (0.84, 3.11) | 10.21 (12.24, 23.21) | 1.09 (0.98, 1.33) | 24.21 (22.69, 27.50) | 2.85 (2.59, 3.46) |

Data are presented as geometric mean (95% confidence interval). CGM, continuous glucose monitoring; GMI, glucose management indicator; TIR, percentage of time in range 3.5 – 7.8 mmol/L; TAR, percentage of time above target range 7.8 mmol/L; TBR, percentage of time below target range 3.5 mmol/L; SD, standard deviation; CV, coefficient of variation; MAGE, mean amplitude of glycemic excursions; GDM, gestational diabetes mellitus based on 2013 WHO criteria; BMI, body mass index. CGM, continuous glucose monitoring; IRGMI, glucose management indicator; TIR, percentage of time in range 3.5 – 7.8 mmol/L; TAR, percentage of time above target range 7.8 mmol/L; TBR, percentage of time below target range 3.5 mmol/L; SD, standard deviation; CV, coefficient of variation; MAGE, mean amplitude of glycemic excursions.

* p < 0.05.
