# Supplementary material for: Early continuous glucose monitoring-derived glycemic patterns are associated with subsequent insulin resistance and gestational diabetes mellitus development during pregnancy
Source: Diabetol Metab Syndr. 2024 Nov 14;16:271. doi: 10.1186/s13098-024-01508-4 (PMC11562738; doi:10.1186/s13098-024-01508-4)
Supplement: Supplementary file 4 — Additional file 4. The predicted 24-hour interstitial glucose levels for women with at least 10 days of CGM readings by (a) IR (GMR 1.12; 95% CI 1.03, 1.21) and (b) GDM status (1.09; 0.99, 1.20) based on the GEE analysis. Red represents the women with IR or GDM and blue represents the women with non-IR or non-GDM. The circle markers and capped vertical lines represent the predicted mean daily glucose levels and the respective 95% CI based on the exponentiated log-transformed hourly median glucose values. Models were adjusted for age, ethnicity, years of education, parity, history of GDM or family history of diabetes, pre-pregnancy body mass index, irregular meal, physical activity, and an interaction term between glycemic status and time. CI confidence intervals, GDM gestational diabetes mellitus based on 2013 World Health Organization criteria, GEE generalized estimating equations, GMR geometrical mean ratio, IR insulin resistance based on HOMA2-IR of at least 1.22. [file 13098_2024_1508_MOESM4_ESM.pptx]

## Slide 1
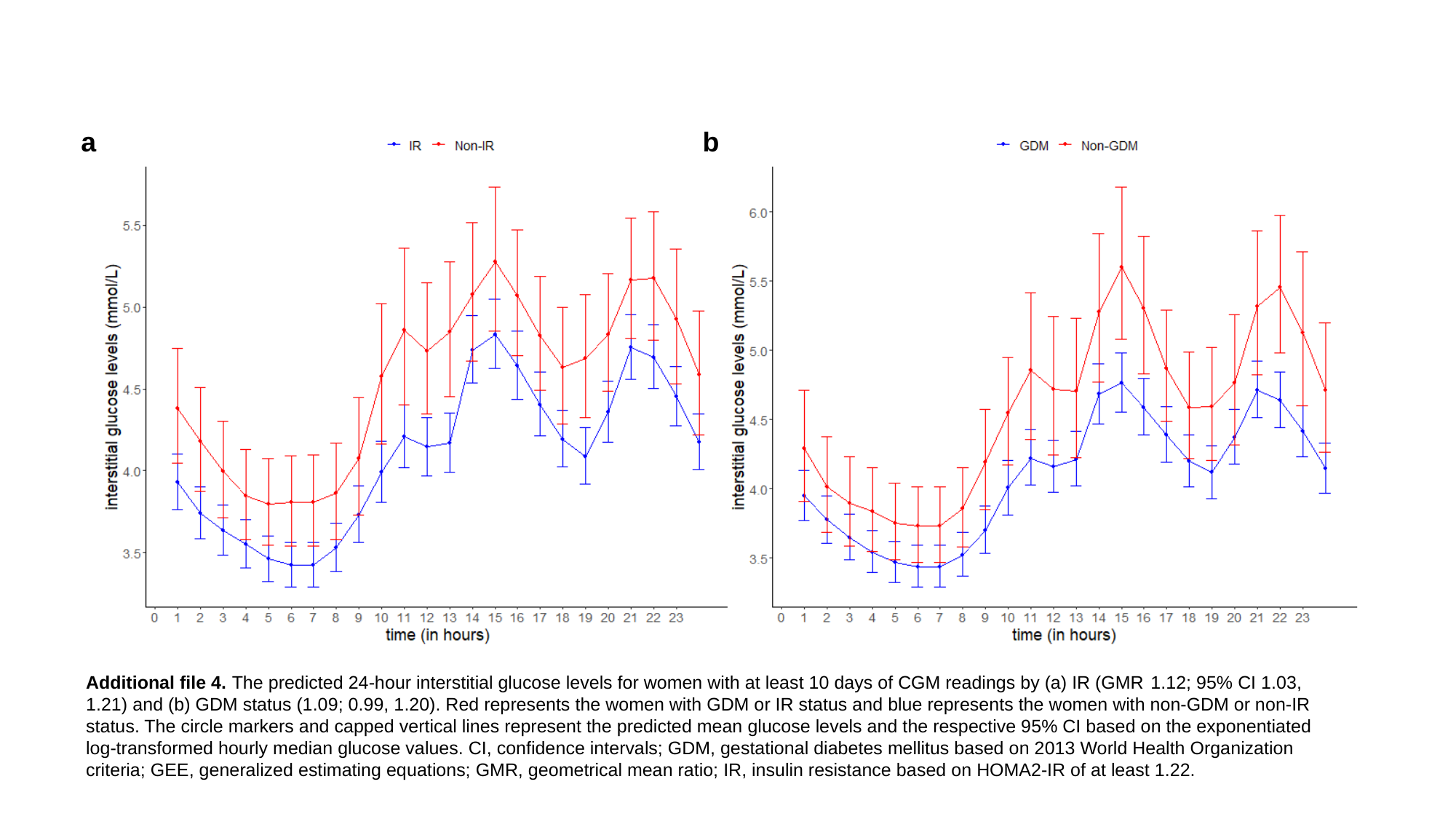

a
b
Additional file 4. The predicted 24-hour interstitial glucose levels for women with at least 10 days of CGM readings by (a) IR (GMR 1.12; 95% CI 1.03, 1.21) and (b) GDM status (1.09; 0.99, 1.20). Red represents the women with GDM or IR status and blue represents the women with non-GDM or non-IR status. The circle markers and capped vertical lines represent the predicted mean glucose levels and the respective 95% CI based on the exponentiated log-transformed hourly median glucose values. CI, confidence intervals; GDM, gestational diabetes mellitus based on 2013 World Health Organization criteria; GEE, generalized estimating equations; GMR, geometrical mean ratio; IR, insulin resistance based on HOMA2-IR of at least 1.22.
